# Regeneration of Bone Defects in a Rabbit Femoral Osteonecrosis Model Using 3D-Printed Poly (Epsilon-Caprolactone)/Nanoparticulate Willemite Composite Scaffolds

**DOI:** 10.3390/ijms221910332

**Published:** 2021-09-25

**Authors:** Latifeh Karimzadeh Bardeei, Ehsan Seyedjafari, Ghamartaj Hossein, Mohammad Nabiuni, Mohammad Hosein Majles Ara, Jochen Salber

**Affiliations:** 1 Developmental Biology Laboratory, Animal Biology Department, School of Biology, College of Science, University of Tehran, Tehran 1417935840, Iran; latifehkarimzadeh@gmail.com; 2 Department of Biotechnology, College of Science, University of Tehran, Tehran 1417935840, Iran; 3 Department of Cellular and Molecular Biology, Faculty of Biological Sciences, Kharazmi University, Tehran 15719-14911, Iran; devbiokharazmi@gmail.com; 4 Photonics Laboratory, Physics Department, Kharazmi University, Tehran 15719-14911, Iran; majlesara@gmail.com; 5 Applied Science Research Centre, Kharazmi University, Tehran 15719-14911, Iran; 6 Salber Laboratory, Centre for Clinical Research, Department of Experimental Surgery, Ruhr-Universität Bochum, 44780 Bochum, Germany; jochen.salber@hotmail.com; 7 Department of Surgery, Universitätsklinikum Knappschaftskrankenhaus Bochum GmbH, 44892 Bochum, Germany

**Keywords:** osteonecrosis, composite, npW, 3D biofabrication, biocompatibility

## Abstract

Steroid-associated osteonecrosis (SAON) is a chronic disease that leads to the destruction and collapse of bone near the joint that is subjected to weight bearing, ultimately resulting in a loss of hip and knee function. Zn^2+^ ions, as an essential trace element, have functional roles in improving the immunophysiological cellular environment, accelerating bone regeneration, and inhibiting biofilm formation. In this study, we reconstruct SAON lesions with a three-dimensional (3D)-a printed composite made of poly (epsilon-caprolactone) (PCL) and nanoparticulate Willemite (npW). Rabbit bone marrow stem cells were used to evaluate the cytocompatibility and osteogenic differentiation capability of the PCL/npW composite scaffolds. The 2-month bone regeneration was assessed by a Micro-computed tomography (micro-CT) scan and the expression of bone regeneration proteins by Western blot. Compared with the neat PCL group, PCL/npW scaffolds exhibited significantly increased cytocompatibility and osteogenic activity. This finding reveals a new concept for the design of a 3D-printed PCL/npW composite-based bone substitute for the early treatment of osteonecrosis defects.

## 1. Introduction

SAON, a common multistage chronic disease, is more prevalent in certain patients < 45 years. The knee is the second most common site of SAON and is characterized by a loss of bone blood circulation, expansion of the bone necrotic area, and subchondral bone collapse [1]. Steroid-associated osteonecrosis of the knee is reported in 10% of all patients diagnosed with osteonecrosis and presents as a bilateral disease in 58–80% of cases. 75% to 90% of osteonecrosis of the knee is highly associated with long term use of glucocorticoids in patients who have undergone organ transplantation and those who receive high doses of glucocorticoids as a life-saving agent for serious infectious diseases such as acquired immune deficiency syndrome (AIDS) and severe acute respiratory syndrome (SARS) or as a disease-modifying drug for chronic autoimmune diseases such as rheumatoid arthritis (RA) and systemic lupus erythematosus (SLE) patients [2,3].

Different treatment modalities (high tibial osteotomy, bone grafting, and core decompression) were used with different limitations, such as invasiveness, comorbidity of the autologous cancellous bone harvest site, a second surgery to remove osteosynthesis material, necrosis of autologous grafts, or lack of biomechanical stability of the alloplastic biomaterial used to fill the defect [4]. Despite the lifelong regenerative and remodeling capacity of the bone, large bone defects and those in the main loading zones of the joint require more treatment intervention [5]. Currently, orthopedic surgeons prefer a biological replacement for younger patients and a permanent and metallic surface replacement for older people [6,7]. In the field of bone tissue engineering, due to the presence of bone marrow stem cells and osteoblasts at the site of injury, emphasis on the osteoconductive properties of the scaffold alone has been crucial; thus, a critical goal is to build osteoinductive scaffolds that can deliver regenerative signals to cells [8,9]. Despite the success of autologous bone grafts in reconstructing and healing bone defects, the limited bone resources in grafts and the disadvantages of the above alternative treatment strategies motivate the design and fabrication of new biologically active biomaterials for bone regeneration [5,10].

This is one of the main goals of bone tissue engineering: the design and fabrication of 3D scaffolds that are not only biocompatible, biodegradable, and specifically porous, but also have a mechanical stability appropriate to bone tissue and additionally possess bioactivity [5,11,12]. Nowadays, various classes of materials are available in the field of biomaterial sciences. Particular attention was made to the use of bioresorbable synthetic and natural polymers and bio glasses or ceramics, as well as their combinations, called composites, in regenerative tissue engineering [9].

Among the synthetic polymers, the linear and aliphatic poly (epsilon-caprolactone) (PCL) is a well-known and frequently used biomaterial used in bone tissue engineering (BTE) [13,14,15,16]. PCL has a melting point of approximately 62 °C and a glass transition temperature Tg of −60 °C and is semi-crystalline at a physiological body temperature (36.5 °C–37.4 °C) [17]. The polymer is available in medical-grade, it is biodegradable and bioresorbable, has a good immunocompatibility, and exhibits excellent technical possibility, especially in the context of modern biofabrication technologies such as electrospinning [18], melt electrowriting (MEW), and bioprinting [19,20,21,22].

Apart from the fact that PCL is easily miscible with other polymers, it can be excellently combined with bioceramic materials. The latter has been added to PCL for several years, and especially recently, in various forms such as nanoparticles or in fiber form, which allows the properties of the PCL to be specifically adjusted. The low degradability rate of PCL under physiological conditions makes it a good choice for long-term tissue engineering applications [16].

One of the major disadvantages of PCL is its lack of functional groups along the aliphatic chains, giving it a hydrophobic character. In addition to the targeted setting of an interconnecting porosity, the hydrophilicity of temporary cell support structures in tissue engineering for complete wetting with cell culture medium in vitro or body fluids in vivo for rapid colonization with migrating cells plays another crucial role in creating a suitable and uniform environment for the host cells in the scaffolds [23]. There are different options to generate hydrophilic surface properties of PCL-based scaffolds by copolymerization, forming blends, PCL composites, or functionalization of PCL scaffolds [24,25].

Willemite (W) is an important silica-based bioceramic in the zinc-silicium biosystem [26]. Older work already indicates that silica-based biomaterials, such as bioglasses, and silica-based bioceramics have an influence on gene expression and protein biosynthesis of osteoblasts [27,28,29]. They also appear to influence juvenile bone development and calcification [26,30,31]. Silicium (Si) is directly related to calcium and occurs in places where calcium levels are relatively low, in a combination similar to hydroxyapatite (HAp), and has also been shown to cause slow calcium deficiency in infants with skeletal growth and deformed skulls. The relationship between silicium and calcium in bone formation has been proven, and its increase in the diet has led to an increase in bone mineralization [32].

Bivalent zinc cations (Zn^2+^) from maternal blood have played a critical role in the long-term development of the fetus through skeletal development. The amount of Zn^2+^ in the bone gradually decreases with age, and therefore Zn^2+^-containing biomaterials can stimulate the activity of bone-forming cells and facilitate the formation of new bone [26,30,31,33]. Most of the Zn^2+^ ions in the body accumulate in the bones and are released during bone resorption. Zn^2+^ acts as an activator of different proteins involved in the ossification process, including the transcription factor associated with runt-related transcription factor 2 (Runx2), collagen type I alpha 1 (Col1A1), and osteoblast-specific transcription factor osterix (Osx) [34,35].

The study aimed to investigate the bone regenerative potential of a novel composite of PCL and nanoparticulate Willemite (npW) in SAON defects of the rabbit distal femur. For this purpose, the npW was first synthesized and analyzed. Different PCL/npW composites with different weight percentages of npW were formulated and analyzed for their in vitro cytocompatibility and property on osteogenic differentiation activity of rabbit bone mesenchymal stem/stromal cells (rBMSCs). Finally, the composite of PCL/5% wt. npW was processed into 3D-printed interconnected porous scaffolds and investigated in the SAON rabbit model.

## 2. Results

### 2.1. Characterization of npW

**Figure 1:**
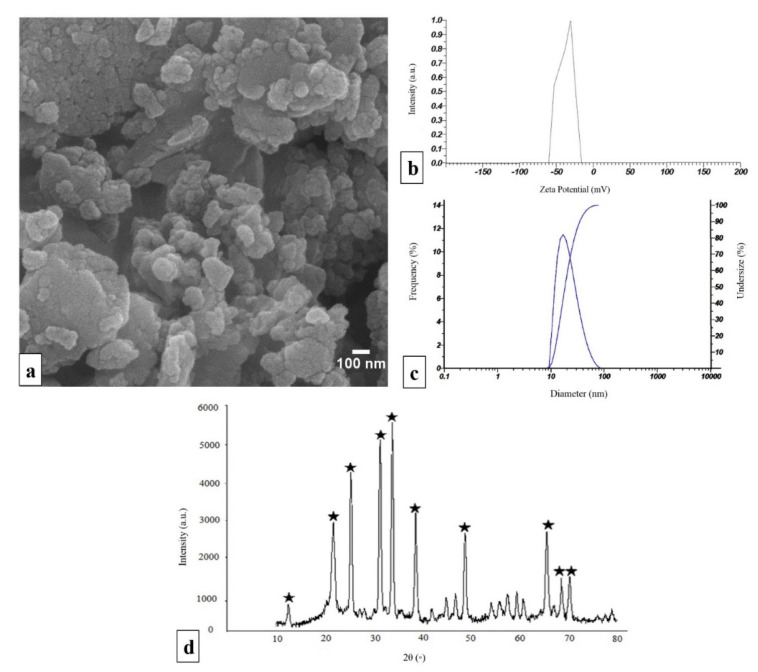
npW characterization. (**a**) Morpho-structural characterization of nanoparticles obtained from SEM microscope, (**b**) particle size distribution in the range of 10 to 100 nm by DLS analysis, (**c**) the zeta potential under the applied analysis conditions resulted in a value of −36 mV, (**d**) XRD spectrum of npW reveals the signals at 2θ values marked with asterisks.

The SEM image shows a section of the synthesized npW bioceramics in powder form. The agglomerated particles have a relatively smooth surface and were rather roundish in shape. Furthermore, the approximate diameter of individual particles can be quantified with values between 20 and 70 nm (Figure 1a)**.** To verify this approximate distribution of values of npW nanoparticle diameters from the scanning electron microscope (SEM) images, the dynamic light scattering (DLS) method was applied. The DLS measurements revealed a particle size distribution from 10 to 100 nm with a maximum of 30 nm (Figure 1b). To be able to make a statement about the stability of dispersions of the npW synthesized here, zeta potential measurements were also carried out. Under the measurement conditions given in the method section, a zeta potential of -36 mV resulted for these npW formulations (Figure 1c). X-ray diffraction (XRD) was also used to study the synthesized npW bioceramics to demonstrate their nanocrystallinity structure and confirm their atomic composition and structure (Figure 1d). The chemical composition of the npW was determined by X-ray fluorescence (XRF) analysis. According to XRF results, in npW nanoparticles (Zn_2_SiO_4_), the weight percentages of the SiO_2_ and ZnO are 36.25% and 59%, respectively.

### 2.2. PCL/npW Composite Scaffolds Characterization

**Figure 2:**
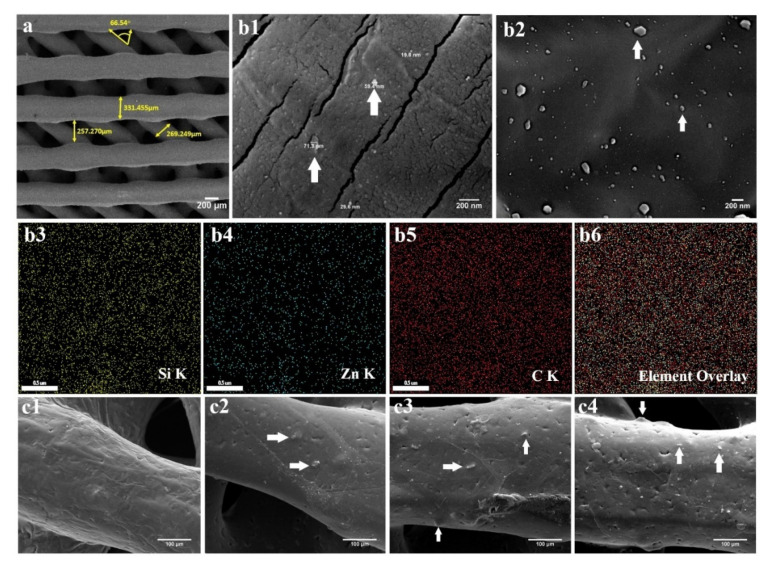
PCL/npW Composite scaffolds characterization. (**a**) SEM microscopy of diameter and orientation of 3D-printed scaffold strands. (**b1**,**b2**) SEM-MAP image showing the distribution of npW nanoparticles in the composite. (**b3**–**b6**) Element surface distribution of Si, Zn, and C and combined view of them by SEM-EDX. SEM image of the surface of a strand of (**c1**) neat PCL, (**c2**) PCL/2.5% wt. npW, (**c3**) PCL/5% wt. npW and (**c4**) PCL/10% wt. npW Nanoparticles embedded in scaffolds are marked with arrows.

SEM imaging showed that the angle between 3D-printed strands of a layer and the next print layer below or above was 60 ± 5°. The diameter of each strand was 400 μm and their distance from each other was 300 μm on average (Figure 2a). The distribution of the npW in the scaffold strands was monitored through SEM/MAP analysis, (Figure 2(b1,b2)). The elemental distribution mappings of Si, C, and Zn in the scaffolds were shown by SEM-electron dispersive X-ray photoelectron spectroscopy (SEM-EDX) (Figure 2(b3–b6)). The composite structure of all 3D-printed scaffolds was revealed by SEM (Figure 2(c1–c4)).

### 2.3. Characterization of PCL/npW Composite Scaffolds

#### 2.3.1. Wettability and Degradation Rate

Water-uptake of neat PCL scaffolds, PCL/2.5% wt. npW, PCL/5% wt. npW and PCL/10% wt. npW was 34%, 38%, 67.43% and 72.20%, respectively. To study the effect of npW on the wetting properties of PCL, contact angle measurements were carried out on neat PCL scaffolds, PCL/2.5% wt. npW, PCL/5% wt. npW and PCL/10% wt. npW. The results of the contact angle analysis using the sessile drop method showed that the contact angle of the pure PCL scaffold was 86°. Contact angle values obtained for PCL/2.5% wt. npW, PCL/5% wt. npW and PCL/10% wt. npW were 93°, 102°, and 108°, respectively.

**Figure 3:**
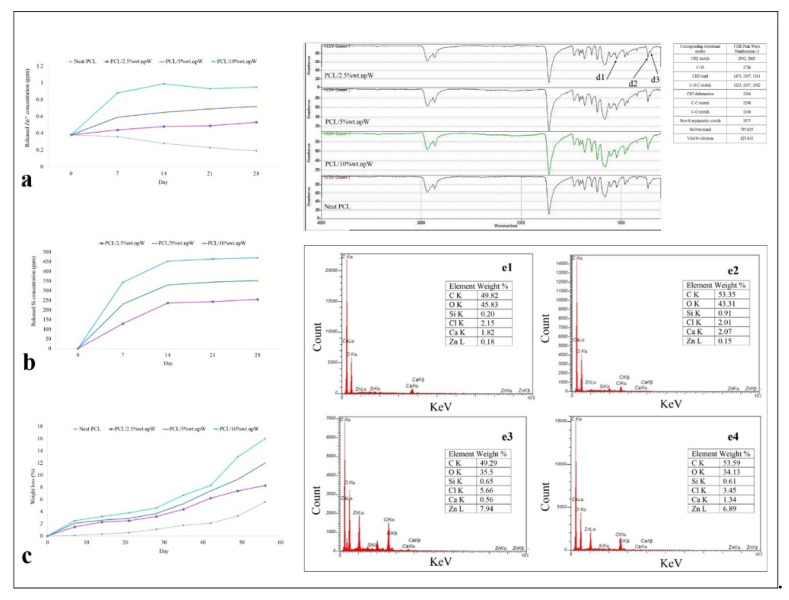
Chemical analysis of the neat PCL and composite scaffolds. (**a**) Zn^2+^ and (**b**) Silicium ion release from PCL/2.5% wt. npW, PCL/5% wt. npW and PCL/10% wt. npW, (**c**) weight loss of scaffolds overtime under physiological conditions. FTIR spectra of all materials: Si-O-Si asymmetric stretching vibrational mode in (**d1**) 1075 cm^−1^, (**d2**) SiO_4_ vibration mode in 825–977 cm^−1^, (**d3**) torsional vibrations modes of Si-O in 707–825 cm^−1^. (**e**) EDX spectra of PCL scaffolds incubated in SBF for (**e1**) 14 and (**e2**) 21 days and PCL/5% wt. npW scaffolds incubated in SBF for (**e3**) 14 and (**e4**) 21 days.

The amount of Zn^2+^ and Silicium released into the DMEM medium at 37 °C, physiological pH and in the presence of 5% CO_2_ over the periods of 7, 14, 21 and 28 days showed that the rate of release of Silicium ions increases steadily over time until a plateau is reached, while the release of Zn^2+^ ions is much slower (Figure 3a,b). The in vitro degradation behavior of PCL, PCL/2.5% wt. npW, PCL/5% wt. npW, and PCL/10% wt. npW was determined by testing the weight loss ratio after immersion in PBS. The results revealed that the weight loss ratio of all the samples increased with incubation time, and the weight loss ratios of PCL/npW samples increased with the increase of the npW content. It was found that the weight loss ratio of PCL/2.5% wt. npW was 8.3%, while that for PCL/5% wt. npW was 12% and that for PCL/10% wt. npW was 16% after 8 weeks (Figure 3c).

#### 2.3.2. Chemical Analysis of the PCL/npW Composite Scaffold

The surface area analyses of the nanoparticles were determined using the brunauer-emmett-teller (BET) method. The surface area of the npW, neat PCL, PCL/2.5% wt. npW, PCL/5% wt. npW and PCL/10% wt. npW, was 3.3558, 1.1744, 2.3503, 2.5672, and 2.5733 m^2^ g^−1^, respectively. Mean pore diameter in the npW, neat PCL, PCL/2.5% wt. npW, PCL/5% wt. npW, and PCL/10% wt. npW were 24.664, 10.6900, 8.8753, 8.4621, and 8.3254 nm, respectively.

The FTIR results demonstrated the absorption peaks at 825–977 cm^−1^ due to the asymmetric stretching vibration of SiO_4_ groups, the Si-O-Zn stretching at 707–840 cm^−1^, Si-O-Si asymmetric stretching vibrational mode in 1075 cm^−1^, torsional vibrations modes of Si-O in 707–825 cm^−1^ (Figure 3(d1–d3)). The results of EDX showed the element weight percentage of C, O, Si, Cl, Ca, and Zn in neat PCL and PCL/5% wt. npW composite incubated 14 and 21 days in simulated body fluid (SBF) at 36.5 °C and pH 7.4 using a sample mass/SBF ratio of 1.5 mg/mL in each plastic vial (Figure 3(e1–e4)).

### 2.4. In Vitro Cytocompatibility

**Figure 4:**
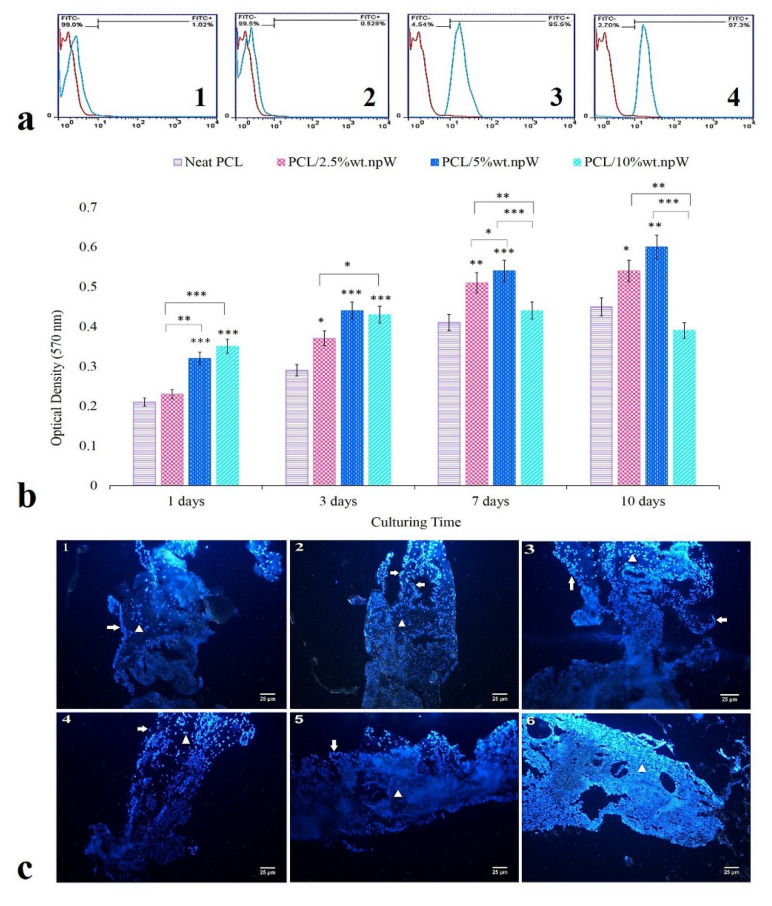
Cytocompatibility analyses of PCL/npW versus neat PCL. (**a1**–**a4**) Characterization of rBMS cells using alpha-SMA, CD44, CD34, and CD45 markers by flow cytometry. (**b**) Viability of rBMSCs on scaffolds at 1, 3, 7, and 10 days of cell culture by MTT assay (**c1**–**c6**). Distribution of rBMSCs in porous PCL/5% wt. npW scaffolds visualized using histological cross-sectioning and DAPI nucleus staining, on 3, 5, 7, 9, 11, 13 days after culture, respectively; the arrows and arrow-heads represent the rBMSCs on the surface and within the scaffold, respectively. Data are shown as mean ± SD. * *p* < 0.05, ** *p* < 0.01, *** *p* < 0.001 compared to neat PCL.

Harvested rBMSCs were positive for: 99% alpha-smooth muscle actin (alpha-SMA), 99.5% CD44, and 2.7% CD34 and 4.54% CD45 (Figure 4(a1–a4)). Cell viability was assessed by subsequent MTT assay at 1, 3, 7, and 10 days of cell culture. We observed a significantly increased cell viability in the PCL/2.5% wt. npW group at 3 (*p* < 0.05), 7 (*p* < 0.01) and 10 (*p* < 0.05) days compared to neat PCL (Figure 4b). In the PCL/5% wt. npW group, the cell viability was significantly increased for 1, 3, 7 (*p* < 0.001), and 10 (*p* < 0.01) days in cell culture compared to neat PCL (Figure 4b). However, cell viability was significantly increased at 1 and 3-day time points for PCL/10% wt. npW group compared to neat PCL (*p* < 0.001) (Figure 4b). The migration and distribution of rBMS cells within the 3D-printed porous scaffolds, which consisted of PCL/5% wt. npW, over a cultivation period of 1 to 13 days, were detected using histological cross-sections and staining of the cell nuclei with DAPI (4′,6-diamidino-2-phenylindole). In the first few days, there was an accumulation of cells on the surface of the scaffold, which migrated into the pores of the scaffold in a time-dependent manner and were accommodated there (Figure 4(c1–c6)). 

**Figure 5:**
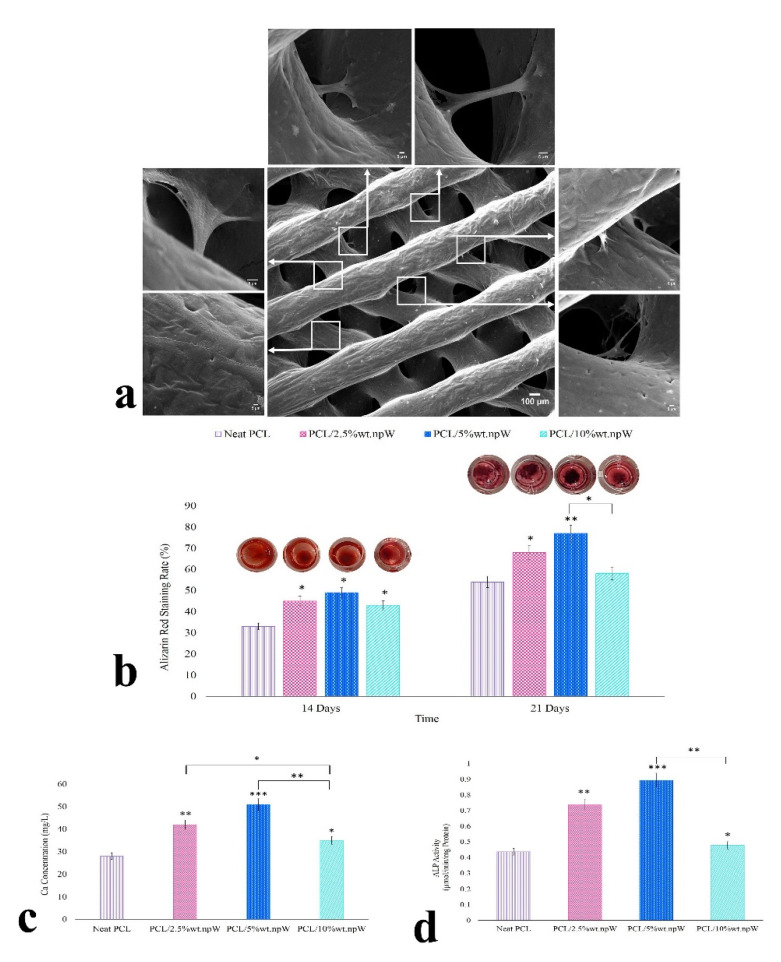
Morphology and differentiation of the rBMSCs in the 3D scaffolds. (**a**) The cell morphology and spreading of rBMSCs cultured on PCL/5% wt. npW. at 7 days post-seeding. (**b**) The photograph of calcium mineral deposits and the amount of calcium deposits were analyzed by Alizarin Red staining assay. (**c**) The quantitative analysis of calcium concentration after 21 days culture under osteogenic medium conditions. (**d**) ALP (Alkaline phosphatase) activity was the quantified concentration after 14 days in culture under osteogenic medium conditions, such as μmol of p-nitrophenol phosphate produced per min and mg protein. Data are shown as mean ± SD. * *p* < 0.05, ** *p* < 0.01, *** *p* < 0.001 compared to neat PCL.

Images obtained by SEM microscopy showed the presence of cells on the PCL/5 wt. npW scaffold surface, as well as within the pores (Figure 5a).

### 2.5. Osteogenic Differentiation

SEM images demonstrated adherence and spreading of the rBMSCs at 7 days post-seeding, not only superficially on the printed ridges of the scaffolds, but also within the pores formed from the ridges of different layers (Figure 5a).

Alizarin Red staining was used for all npW samples against the PCL-only control after 14-day and 21-day rBMSC cultivation concerning Ca deposition. Thereby, all three npW-modified samples showed no significant difference in Ca deposition among each other, but a significant increase compared to pure PCL after 14 days. After a cultivation period of 21 days, the PCL composite samples with 2.5% wt. npW and 5% wt. npW showed a further significant increase in Ca deposition compared to pure PCL. However, this was not the case for the PCL/10% wt. npW (Figure 5b).

In addition to the semi-quantitative Alizarin Red staining at cultivation times 14 and 21 days, another quantitative Ca expression assay was used to verify cultivation times 21 days data. This revealed completely analogous trends among the composites and significant increases in Ca expression and deposition compared to the unmodified PCL. The decrease in the amount of Ca produced and deposited in the PCL/10% wt. npW composite was striking (Figure 5c).

The ALP activity showed for the already named samples in comparison to pure PCL for the cultivation times 14 days equal tendencies among each other and significances compared to the control (Figure 5d).

### 2.6. The Effect of PCL/npW Scaffold on the Reconstruction of SAON Lesion in the Rabbit Femur

**Figure 6:**
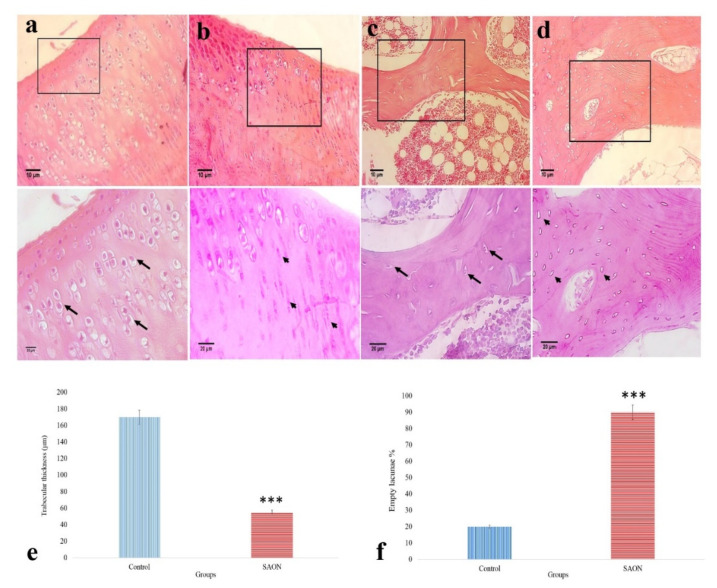
Histological confirmation of SAON induction in the rabbit. Articular cartilage cell morphology in (**a**) control and (**b**) SAON group. Subchondral bone in (**c**) control and (**d**) SAON group. Arrows and arrowheads indicate healthy and damaged cartilage cells as well as bone lacunae containing osteocytes in articular cartilage and subchondral bone, respectively. (**e**,**f**) The trabecular bone thickness (µm) and the empty lacuna in the control and SAON group. Data are shown as mean ± SD. *** *p* < 0.001 compared to control.

At first, we evaluated the histology of knee articular cartilage (Figure 6a,b) and subchondral bone (Figure 6c,d) at two months post-steroid injection compared to control. The typical osteonecrosis in the SAON group is characterized by the reduced average thickness of bone trabeculae 55 µm versus 170 µm in the control group (Figure 6e) and the presence of a higher percentage of empty lacunae (88% versus 20% in the control group) (Figure 6f).

**Figure 7:**
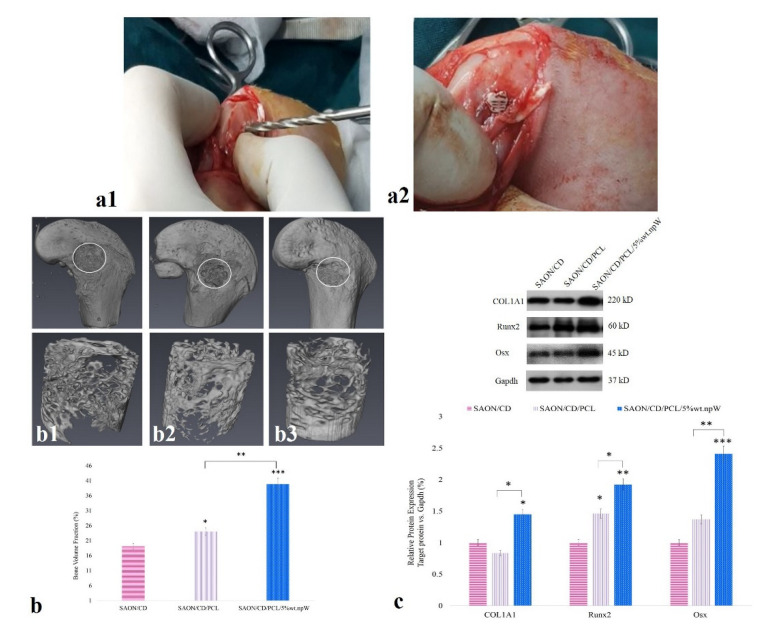
In vivo bone regeneration. (**a1**) Creation of 10 mm diameter CD. (**a2**) Press-fitted the neat PCL and PCL/5% wt. npW scaffold into the bone CD tunnel. (**b1**–**b3**) Micro-CT analysis of SAON/CD, SAON/CD/neat PCL, and CD/PCL/5% wt. npW groups at 2 months post-steroid injection. (**c**) Western blots were used to determine the expression of the proteins Col1A1, Runx2, and Osx in the tissue of the distal femur normalized to Gapdh expression. Results showing mean ± SD of at least three independent experiments. * *p* < 0.05; ** *p* < 0.01; *** *p* < 0.001 compared to SAON/CD.

Next, to evaluate the in vivo osteogenesis potential of scaffolds containing npW, the cylindrical scaffolds containing 5% wt. npW were inserted into a 10 mm × 3 mm hole in the distal femur of animals with confirmed osteonecrosis (Figure 7(a1,a2)). Micro-CT was used to determine the quality and quantity of ossification capacity in the distal part of the rabbit femur with SAON. The results showed a significantly increased bone volume fraction (BVF) in SAON/CD/neat PCL (*p* < 0.05) and SAON/CD/PCL/5% wt. npW (*p* < 0.001) groups compared to the SAON/CD group (Figure 7(b1–b3)).

### 2.7. PCL/npW Scaffold Effect on Col1A1, Runx2, and Osx

Ossification in the SAON-induced animals was assessed by quantifying the expression levels of ossification markers: Col1A1, Runx2, and Osx in the rabbit femur (Figure 7c). Col1A1 and Osx expression levels were significantly increased by 1.5 and 2.4 fold, respectively in SAON/CD/PCL/5% wt. npW group compared to the SAON/ CD group (*p* < 0.01). Runx2 in expression levels were significantly increased in both SAON/CD/neat PCL (1.5 fold) and SAON/CD/PCL/5% wt. npW (1.4 fold) groups compared to SAON/CD group (*p* < 0.05 and *p* < 0.01, respectively).

## 3. Discussion

Regeneration of osteonecrotic bone defects using a tissue engineering approach is seen as a potential alternative to the traditional use of bone grafts, as they are available in an unlimited supply and there is no fear of disease transmission, additional donor site morbidity, immune rejection, or pathogen transfer. For these reasons, there is currently an extremely high interest in functional biomimetic biomaterials [5,6,36]. To achieve this goal, biocompatible scaffolds are developed that mimic the architecture of the extracellular matrix of the target bone as closely as possible to achieve the induction of new functional bone tissue [37]. Based on CT and magnetic resonance imaging (MRI) data sets, patient-specific, defect-analog computer models can be created, which are converted into sophisticated geometries with internal structures for patient-specific defect coverage using increasingly precise 3D printing technology in a layer-by-layer manufacturing process [34]. Utilization of porous scaffolds as a resorbable and biomimetic implant/filler can enhance the efficacy of CD in SAON patients and can strengthen the mechanical and structural support while facilitating bone regeneration and prevent the further bone collapse of the load-bearing joints.

As the bone tissue is composed of inorganic and organic components, composite materials represent attractive candidates for bone regeneration medicine. Synthetic polymer-ceramic composites capitalize the advantages of each of its components and demonstrate osteoconductivity that is superior to their pure polymer counterparts [38]. The current study represents a novel polymer-ceramic composite that offers a promising option for bone tissue engineering applications. The polymer-ceramic composite is based on the specific strategies that have been proposed in long-term bioengineering, namely scaffold surface modulation, topography, incorporation of bioactive elements, and their release rating. Willemite ceramic nanoparticles (Zn_2_SiO_4_) were used as an important bioactive ceramic in silica-based bioceramics to modify PCL hydrophobicity and to induce bone regeneration [30,31,33,39]. The essential role of Zn^2+^ as a trace ion in the development of the skeletal system has been proven [40,41].

In this study, npW nanoparticles were synthesized by a solid-state reaction method and the XRD results showed that the sintering of ZnO and SiO_2_ powder mixtures at a temperature of 1250 °C produces stable crystalline npW nanoparticles and this crystalline phase was also visible in the composites with PCL. The fabrication temperature of ceramic nanoparticles is very critical in creating the crystalline phase. In addition to temperature, Zn^2+^ also promotes the crystallization of SiO_2_ in Wollastonite (CaSiO_3_), Hardystonite (Ca_2_ZnSi_2_O_7_), 58S, and 45S bioactive glasses and it was orchestrated with the amount of ZnO [42,43,44]. Since the crystallinity of the polymer did not change much during the processing by bio-printing [45], the crystallinity of the PCL/npW scaffolds can be attributed to the nanoparticles’ crystallinity.

Estimating the stability of npW nanoparticles was one of the essential issues addressed in our study. Zeta potential is a very important tool to understand the surface charge and the stability (prevention of agglomeration) of nanoparticles within the PCL phase and after release [46,47]. Here, we showed by mapping analysis that Si, C, and Zn elements were homogeneously distributed in the printed struts of the scaffolds without showing agglomerated nanoparticles. This was confirmed by -36 mV zeta potential, indicating the relative stability of npW nanoparticles.

Blending the npW with PCL provided both an enhancement of surface roughness and a continuous and slow release at the bone required concentrations [42]. The SEM-MAP results in this study confirmed a homogeneous distribution of nanoparticles and thus Si, C, and Zn elements on and within the scaffold struts made of PCL/npW composites. During the degradation of the polymer scaffold nanoparticles embedded within the scaffold, the struts got exposed to the environment, were released, and could interact chemically and biologically with media, cells, or tissue. This thus resulted in biologically active composites of nanoparticulate Willemite and PCL, which could be confirmed by the following in vitro and in vivo results.

The osteoconductive role of Zn^2+^ on osteoblasts and mesenchymal stem/stromal cells is highly concentration-dependent [34,48]. The survival and proliferation of mesenchymal stem/stromal cells or MC3T3-E1 accrue in the range of 8 to 12 μM Zn^2+^ [48,49]. However, higher concentrations (15 μM and above) of Zn^2+^ disrupt the balance of Zn^2+^ transport on both sides of the membrane by the Zrt- and Irt-like protein (ZIP) and by the zinc transporter (ZnT), leading to excessive accumulation of intracellular Zn^2+^, inhibition of osteogenic differentiation of rBMSCs, increased intracellular ROS production and finally apoptosis [50]. Moreover, ZnO nanoparticles have been reported to be able to release more of this ion as compared to ZnCl_2_, which is due to their nanoparticle nature leading to higher cytotoxicity [48,51]. This may explain our observation here that cell viability was reduced with PCL/10% wt. npW compared to a lower concentration of npW. Mesoporous silica possesses a stable Si-O-Si network structure, which gives the flexibility for doping metal elements into the mesoporous silica framework by partially substituting Si sites and forming an Si-O-M bond (M=Ca, Mg, and Zn). The Si-O-Zn bond is more stable than the Zn-O-Zn bond, thus guaranteeing the sustained release of Zn^2+^ from nanoparticles and its long-term stability [34]. This is in line with our results here, which showed that mesoporous npW have a high tendency to release Zn^2+^ due to their minimal size and high specific surface, as revealed by our DLS and BET results. Moreover, PCL substrate mediates the controlled release of Zn^2+^ from our PCL/npW scaffold. It should be emphasized that the slow release of Zn^2+^ in the current study may be due to the placement of nanoparticles in the low-rate biodegradable scaffold and the presence of Zn^2+^ in the silica network. Moreover, here, the rate of release of Silicium ions regularly increases with increasing treatment time, while the release of Zn^2+^ ions occurs much more slowly than the release of Silicium.

Here, we showed that water absorption and wettability of scaffolds with npW was higher than the neat PCL scaffold. This might be related to the high porosity and roughness of the PCL/npW scaffold compared to pure PCL. Furthermore, it has been reported that osteoblasts preferentially adhere to the surface as they can provide the necessary structural sites for HAp attachment [52,53,54].

The results of 14 and 21-day immersion of the PCL and PCL/5% wt. npW in SBF showed that Zn^2+^ in bioactive glass delays the formation of HAp nuclei in the initial stage (14 days) of SBF soaking. This is due to the absorbance of Zn^2+^ ions at the sites of active Hap, which inhibits further HAp form and growth. Accordingly, several studies reported that the size of HAp nuclei in Zn^2+^-containing glass is more significant than that of Zn^2+^-free glass because each (few) HA nucleation sites had a better chance of absorbing Ca^2+^ and PO_4_^3-^ in SBF [55,56]. On the other hand, Silicium increases the electronegative surface and is a suitable microstructure for nucleation and apatite formation sites [57,58]. In our report here, as the soaking time increased to 21 days, the composite surface was gradually covered by a layer of HAp, and the diffusion of Zn^2+^ from the composite into the solution became more complex than the diffusion of calcium due to the higher Zn-O bond energy (180 kJ/mol) compared with the Ca-O bond (110 kJ/mol). Our EDX analysis here showed that Zn^2+^ percentage in PCL/5% wt. npW were comparable at 14 and 21 days SBF soaking and Ca^+2^ deposit increased. In agreement with this finding here, it has been reported that the inhibitory effect of Zn^2+^ ions on HAp formation was reduced in a time-dependent manner [42].

The optimal pore size used to cover most cellular behaviors, i.e., osteoblast attachment, growth, differentiation, and tissue vessels in vivo in porous HAp scaffolds with spherical pores, is approximately 300–400 μm [59,60]. Accordingly, here we used an interconnected porous composite printed scaffold with a pore size of 300 to 400 μm, able to stimulate successful cellular behaviors and a bone regeneration response.

This was confirmed with the SEM images and DAPI staining, showing a proper interaction of the cell with the surface of the strands, the proper expansion of the cells on them, and their migration from the scaffold surface to the inner cavities.

The cells try to reduce their surface energy to the lowest possible level by reaching the most stable state in the corners of the pores to have more contact with other cells. At the corners of the pores, a slight angle of 90° created a suitable environment with a stable state, while the cells in the center of the pores have the highest energy level and are in an unstable state [21,61]. Tri-angular pores induce osteogenic differentiation and ALP activity, while hexagonal and rectangular pores showed the highest cell proliferation [62]. Therefore, here, we used the pattern of triangular pore geometry because the triangular and concave angles provided the best state of the cells to interact together and to minimize the remaining energy of the cells.

Zn^2+^, Ca^2+^, and cAMP regulate mitogenic signaling and other types of intracellular signaling pathways as secondary messengers and sometimes synergistically stimulated DNA synthesis and mitogenic signaling in mouse fibroblasts [35,63,64]. Zreiqat et al. showed that surface chemical modification of Ti-6Al-4V with essential divalent elements such as magnesium and Zn^2+^ enhances the activity of bone cells due to changes in cytoskeleton organization, especially microfilament structures [65]. Another study showed that cells cultured on Ca_2_ZnSi_2_O_7_ had grown steadily over time compared to cells cultured on CaSiO_3_, with higher proliferation rates [51]. These studies may suggest that npW could influence mitogenic signaling pathways.

Bone matrix maturation and mineralization have been identified as the key markers of osteogenic differentiation. Fielding et al. showed that magnesium and Zn^2+^ as a mitogenic factor keep the cells in the proliferative state for up to 11 days. While magnesium is not able to induce differentiation, Zn^2+^ and Si could promote osteogenic differentiation. Zn^2+^ can increase ALP activity and DNA content in bone tissue. On the other hand, Zn^2+^ is a known cofactor required for ALP activity. This important enzyme in bone formation can incorporate up to four Zn^2+^ per molecule. ALP itself is a Zn^2+^ metalloenzyme and contains two molecules of Zn^2+^, which if the Zn^2+^ is removed, reduces the activity of this enzyme and leads to a negative regulation of the extracellular matrix mineralization through the inhibition of ALP activity in osteoblasts [51]. Animal studies have also shown an increased skeletal ALP activity in the diaphysis of the femur and tibia of mice fed with a Zn^2+^-rich diet [66]. In line with these reports, our study here showed that the ALP activity of cells cultured on PCL/npW composite scaffolds was significantly higher than on controls.

Moreover, a higher Ca^+2^ accumulation was observed in PCL/2.5% wt. npW and PCL/5% wt. npW groups compared to neat PCL and PCL/10% wt. npW-treated groups. Similarly, higher Ca^+2^ concentration nodules were formed in a time-dependent manner with PCL/2.5% wt. npW and PCL/5% wt. npW, as revealed by an Arsenazo III-based assay. By far, it seems that the presence of a high amount of Zn^2+^ interferes with the capture of Ca^2+^ (Ca cluster forming) and/or inhibited calcium phosphate nucleation [67].

Hence, in the current study, calcium was evaluated in two ways: 10 staining of unstable calcium clusters and crystallized calcium phosphate accumulations by alizarin red staining and a quantitative measuring of total calcium including intracellular calcium and above-mentioned calcium. We showed here that the amount of the clustered and precipitated calcium is less than the amount of total calcium at 21 days, which may indicate that Zn^2+^ competed with calcium in absorption at negative charge positions. This is further supported by the fact that Zn^2+^ ions exhibited an ionic radius significantly smaller than Ca^2+^ and easily enters the calcium phosphate pronuclei structure, distorted it, and created a structural mismatch that prevents further apatite crystal growth (de Araujo Bastos Santana et al., 2021).

Here we showed that PCL/npW scaffolds could promote osteogenic differentiation as revealed by increased levels of Runx2 and Osx transcription factors and a higher synthesis of proteins such as Col1A1 and ALP. Runx2 has a stimulatory role in the differentiation of mesenchymal stem/stromal cells and precursors of osteochondrogenetics and pre-osteogenesis [68,69]. Zn^2+^ is involved in Runx2 expression, which is the first determinant of osteogenic differentiation, and its downstream gene, Osx, is itself a transcription factor containing the zinc finger pattern [57]. Correspondingly, the commitment of MSCs to osteoblast differentiation is dependent upon lineage-specific transcription factors, such as Runx2 and Osx [70]. Osx is specifically expressed in osteoblasts and osteocytes, albeit at lower levels, and is involved in osteoblast differentiation, maturation, and activity. Osx acts as a molecular switch for the formation of an active chromatin state during osteoblast differentiation. The essential role of Osx in osteoblast differentiation is attributed to its ability to regulate the expression of various osteoblast markers such as Col1A1 [70]. Moreover, Runx2 and Osx synergistically regulate the transcription of osteogenesis-related genes through upregulation of ZIP1 expression, which further leads to the induction of Zn^2+^ influx, contributing to a positive feed-forward Zn^2+^ -Runx2/Osx-ZIP1 regulation loop during osteogenic differentiation [35]. Moreover, the effect of Zn^2+^ on Osx gene and protein expression in MC3T3-E1 cell culture at an early stage of osteoblast differentiation was highly time- and dose-dependent [70]. These studies are in line with our findings here, showing higher expression levels of Runx2 and Osx proteins in the SAON/CD/PCL/5% wt. npW compared to SAON/CD and SAON/CD/neat PCL groups.

Col1a1 protein plays a critical role in producing minerals, collagen in bone combines with calcium crystals and forms a complicated structure, so its high expression leads to biomineralization [71]. We showed here higher Col1a1 protein expression levels in SAON/CD//PCL/5% wt. npW compared to the SAON/CD/ neat PCL and SAON/CD groups.

## 4. Material and Methods

### 4.1. Materials and Experimental Reagents

Experimental reagents and equipment were obtained as follows: PCL with number average molecular weight (M_n_) 80,000 and protease inhibitor cocktail (Sigma Aldrich, Saint Louis, MO, USA), fetal bovine serum (FBS), basic medium DMEM/F12, and phosphate-buffered saline (PBS) (Gibco, Grand Island, NY, USA), Silicium dioxide (SiO_2_) nanopowder, 5–20 nm particle size and Zinc oxide (ZnO) nanopowder, <100 nm particle size, alizarin red (Cyagen, Santa Clara, CA, USA), primary antibodies against runx2, and Col1A1 (Santa Cruz, Dallas, TX, USA), primary antibodies against Osx (Abcam, Cambridge, UK), and secondary antibody (Bethyle Laboratories, Inc., Montgomery, TX, USA), NP40 Lysis buffer and polyvinylidene fluoride (PVDF) (Invitrogen, Waltham, MA, USA), phenylmethylsulfonyl fluoride (PMSF) and other reagents (Merck, Darmstadt, Germany).

### 4.2. Preparation of npW

npW bioceramic was prepared according to our published protocol [33]. Briefly, 26.96% *w*/*w* SiO_2_ and 73.04% *w*/*w* ZnO as raw materials were mixed in a ball mill (Biobase Biozone, Jinan, China) for 10 h to assist mechanical activation of the ZnO and SiO_2_ powders and increases their surface area. During sintering at 1250 °C in an electric furnace (Yaran Behgozin Parsa, Iran), a new phase, npW (Zn_2_SiO_4_), starts to nucleate and grow at the contacts between SiO_2_ and ZnO particles. An SEM (FEI ESEM QUANTA 200, Hillsboro, OR, USA) was used to examine the shape and appearance of the particles produced and to estimate their relative size. Particle size, zeta potential, and nanoparticle stability of the samples were analyzed by DLS (Malvern Panalytical, Malvern, UK) using a physiological saline solution (pH 7.4). XRD (Bruker AXS, Karlsruhe, Germany) was used for phase identification of nanoparticles using Cu Kα radiation at = 0.154 nm, operating at 40 kV and 40 mA as a radiation source to generate diffraction peaks from the sample within a 2θ angle range from 10° to 80°. The elemental analysis of the generated compound was performed using the XRF spectrometry (Spectro Midex, Kleve, Germany). The electrode voltage was 3.4 V, the conductivity was 0.119 mS/cm, the dispersion medium viscosity was 0.892 mPs, and the temperature of the holder was 25.2 °C.

### 4.3. Manufacturing of the 3D Interconnected Porous PCL/npW Composite Scaffolds

In the current study, for preparing PCL/npW composite, PCL pellets dissolved in chloroform (10% *w*/*v*) and npW nanopowder were added at mass fractions of 2.5% wt., 5% wt., and 10% wt. to produce PCL/2.5% wt. npW, PCL/5% wt. npW and PCL/10% wt. npW scaffolds. They were printed by 3D-printer (3DPL bioprinter N2, Iran) in a disc shape (9 mm × 1 mm) for in vitro use and a cylinder shape (3 mm × 10 mm) for in vivo experiments. The printing parameters of the experimental scaffolds were as follows: printing temperature 75 °C, gas pressure 3.38 bar, printing rate 6 mm/min. Using micro-CT, a non-destructive 3D inspection of the 3D-printed scaffolds was carried out with a high spatial resolution. SEM was applied to analyze specific important details, such as strut diameter, pore size, layer thickness, and surface topomorphology. SEM combined with EDX was used for chemical surface mapping.

### 4.4. Characterization of PCL/npW Composites

#### The Water Uptake and Wettability

The wettability of the different 3D-printed PCL/npW composites was evaluated by applying static water contact angle (WCA) measurements in sessile drop mode. The measurements were carried out by using an OCA 15 Pro Goniometer (DataPhysics, Filderstadt, Germany). Deionized water droplets of 4 µL were deposited via a motorized syringe at a velocity of 1 µL/s. Five measurements were taken from each sample at different surface positions. A high-speed frame camera was used to capture the drop shape. All measurements were performed in the 20 s after adding the drop. The percentage of equilibrium water uptake ability of the porous scaffolds was determined using the following formula: [Water Uptake% = W_w_ − W_d_/ W_d_ × 100], W_w_ and W_d_ are the weight of wet and dry samples, respectively.

### 4.5. Ion Release and Degradation Rate

The porous PCL/npW scaffolds were placed in tubes, submerged in DMEM medium containing 10% FBS, and placed in an incubator at 37 °C and 5% CO_2_. The pH and Zn and Si ions concentrations of the soaking solution were measured at 3, 7, 14, 21, and 28 days using a pH meter (Mettler Toledo, Greifensee, Switzerland) and an inductively coupled plasma mass spectrometer (ICP-MS) (Varian Vista MPX, Markham, ON, Canada). The mass of the scaffolds was measured as a function of time under equilibrium conditions to determine the mass loss of the scaffolds due to degradation. The degradation rate was determined as a weight loss percentage at each time point using the following equation: [Weight Loss% = W_deg_ − W_int_/ W_int_ × 100], where W_int_ and W_deg_ are the initial weight and weight after a specific time of degradation, respectively.

#### Brunauer Emmett-Teller (BET) and FTIR Analysis

FTIR measurements of PCL/npW composites were performed by Nicolet 5700 (Thermo Nicolet, Waltham, MA, USA) in transmittance mode at room temperature from 400 to 4000 cm^−1^ with a resolution of 4 cm^−1^. For analyzing surface area and pore dimension of the samples by the BET method (ASAP 2020, Micromeritics Instruments, Norcross, GA, USA), npW were degassed at 180 °C for 6 h and PCL/npW composites were degassed at room temperature for 24 h before the actual measurements. The BET-specific surface area was determined by the multipoint BET method using the adsorption data in the relative pressure (P/Po) range of 0.01–0.99 and 0.05–0.3 (kPa/kPa) for npW and PCL/npW composites, respectively.

### 4.6. Evaluation of the Mineralization Capacity (SEM-EDX)

HAp-deposition ability of the neat PCL and PCL/5% wt. npW scaffolds were investigated based on the methods described previously [72]. Briefly, the PCL/npW scaffolds were immersed in individual test plastic vials containing SBF at 36.5 °C using a sample mass/SBF ratio of 1.5 mg/mL in each vial. The composition of the SBF solution was similar to the ionic composition of human blood plasma: 7.9949 g of NaCl, 0.2235 g of KCl, 0.1470 g of K_2_HPO_4_, 0.3528 g of NaHCO_3_, 0.071g of Na_2_SO_4_, 0.2775 g of CaCl_2_, and 0.305 g of MgCl_2_·6H_2_O in 1000 mL of demineralized H_2_O. The pH was adjusted to 7.4 with 50 mM tris-hydroxymethyl aminomethane (Tris) and 45 mm hydrochloric acid (HCl) at 36.5 °C. After 14 and 21 days, SEM-EDX analysis was performed on the neat PCL and PCL/npW scaffolds, which were incubated in SBF.

### 4.7. In Vitro Evaluation

#### Extraction and Culture of rBMSCs

We extracted rBMSCs from the femurs of 4-week-old male New Zealand white rabbits (*Oryctolagus cuniculus*) by a washing and aspiration procedure with DMEM/F12 medium supplemented with 20% FBS and 1% Pen/Strep antibiotics from both ends of the exarticulated femurs. All syringe contents were transferred to the bottom of a 25 cm^3^ cell culture flask and cultured at 37 °C, with 5% CO_2_ and 95% humidity overnight. The media was changed every 2 to 3 days until the blood and hematopoietic cells were removed entirely. rBMSCs remain mostly spindle-shaped and adhere to the bottom of the flask and are passaged at 70% confluency. After three passages, the isolated cell fraction was identified through means of flow cytometry (BD FACSAria, Biosciences, San Francisco, CA, USA) as CD90+/CD44+ and CD34−/ alpha-SMA-cells and thus as rBMSCs.

### 4.8. Investigating the Cytocompatibility of Scaffolds

Cell viability was assessed with a MTT (3-[4, 5-dimethylthiazol-2-yl]-2, 5-diphenyl tetrazolium bromide) reduction test. For this purpose, 20,000 rBMSCs were added to each well of a 48-well tissue culture plate containing the disc shape PCL/npW scaffolds at standard cell culture conditions. Cytocompatibility evaluation was performed at 1, 3, 7, and 10 days post-seeding. At the end of the incubation period, 500 μL of media was replaced by MTT solution (500 μL solution 12 mm). The cells in direct contact with the biomaterial samples were incubated for 4 h and formazan crystals were solubilized using DMSO and the optical density was assessed using a microplate reader (Stat Fax 2100, USA) at 570 wavelengths.

### 4.9. Evaluation of Cell Distribution in Scaffolds Using SEM

To prepare the rBMSCs–PCL/5% wt. npW composite constructs for SEM analysis, the scaffolds were first fixed with 3% glutaraldehyde solution at room temperature for 30 min, then washed twice with PBS, and dehydrated in a graded series of aqueous ethanol solutions and ethanol, respectively (50%, 70%, 80%, 90%, and 100%) and afterwards were air-dried. Then samples were sputtered with a platinum layer in the nm range by a Physical Vapor Deposition (PVD) system (CoXEM, Daejeon, Korea).

### 4.10. Evaluation of Osteogenic Differentiation Effect of PCL/npW Scaffolds

Alizarin Red staining was used to identify calcium deposition and to evaluate the osteoblastic differentiation ability of rBMSCs in the presence of PCL/npW scaffolds. A total of 35,000 rBMSCs were cultured on scaffolds in wells of a 48-well tissue culture plate. After one day, the scaffolds and cells were gently washed, and the culture medium was changed to osteogenic maintenance medium containing 10 mm β-glycerophosphate, 0.2 mm ascorbic acid, and 10 nm dexamethasone for 21 days. The osteogenic medium was changed regularly every 4 days. On 7, 14, and 21 days, the culture medium was removed, the cells were fixed with 4% formaldehyde followed by the addition of 2% Alizarin Red dye solution for 15 min at room temperature and was washed four times with 4mL dH_2_O while shaking for 5 min. For quantification of staining, the plate was incubated at room temperature for 30 min 10% (*v*/*v*) acetic acid, after vortexing for the 30 s, heated to exactly 85 °C for 10 min, then centrifuged at 12,000 rpm for 15 min. For neutralizing the acid, 10% (*v*/*v*) ammonium hydroxide was added and read at 405 nm.

### 4.11. Evaluation of Alkaline Phosphatase Activity

To measure the alkaline phosphatase (ALP) activity of the cells that were in direct contact with the PCL/npW scaffolds, total protein was extracted from these adhered cells using 0.05% Triton and 10 mm Tris-HCL, pH 7.5 centrifuged in 12,000 rpm for 10 min. ALP activity was then assessed using a commercial kit (Biorexfars, Fars, Iran) containing magnesium sulfate, diethanolamine, and p-nitrophenyl phosphate. ALP activity was normalized to total protein content (µmol/min/mg protein).

### 4.12. Calcium Content Assay

A calcium assay kit (Biorexfars, Fars, Iran) was used to analyze and determine the calcium in the studied samples. The scaffolds were homogenized with 0.6M HCl and shaken at 40 °C for 4 h. 10 µL of the extract was added to 100 µm Arsenazo III in 90 µL of ddH_2_O. After 10 min at room temperature, the absorbance of the samples was measured at 595 nm.

### 4.13. In Vivo Evaluation

#### Animals

Fifteen male *Oryctolagus cuniculus* rabbits, two months old, weighing approximately 2 kg, were used for in vivo studies. The animals were kept in special cages with free access to water and dry food. The animal care laboratory was set with a cycle of 12 h of light and 12 h of regular darkness and a temperature of 22 °C ± 2 and a humidity of 50%. All ethical considerations were taken into account following the Helsinki Convention and the observance of animal rights, and experiments were performed after the approval of the Ethics Committee of the University of Tehran (Ethical Code: 61771/17D/6;1398.04.17).

### 4.14. Induction of Osteonecrosis of the Femur in Animals

An SAON model was established according to a previous protocol [73]. Briefly, 24 h after one injection of 10 mg/kg of lipopolysaccharide (LPS) intravenously, three injections of 20 mg/kg of methylprednisolone (MPS) were given every 24 h intramuscularly. Eight weeks after the last MPS injection, the control and SAON animals were sacrificed, and the distal femur parts were removed. Each sample was placed in a small glass container. First, to soften the bone tissue, the samples were placed in 5% nitric acid solution for 14 h and then for fixation in 10% formalin solution for one week. After dehydration and clearing, the sample was placed in melted paraffin to remove all dehydrating and clearing solvents. The bone/paraffin block was fabricated and sliced into 7 μm sections. Hematoxylin and Eosin staining was performed to evaluate the percentage of empty lacunae of osteocytes, and the trabecular diameter of femoral spongy bone compare to control rabbits.

All SAON animals were divided into three study groups:

Group I: SAON animal undergoing core decompression (CD) surgery (SAON/CD, *n* = 5).
Group II: SAON animal undergoing CD surgery and implantation of neat PCL scaffold for eight weeks (SAON/CD/neat PCL, *n* = 5).
Group III: SAON animal undergoing CD surgery and implantation of PCL/5% wt. npW for eight weeks (SAON/CD/PCL/5% wt. npW, *n* = 5).

### 4.15. Surgical Procedure

The animals were anesthetized with a combination of 10% ketamine (80 mg/kg body weight) and 5% pentobarbital (1 g/kg body weight) two months after SAON induction. To perform CD surgery, a drill channel with a depth of 1 cm was created with a 3 mm drill from the medial to the lateral distal femur. After removing the bony fragments from the drill channel by rinsing them with physiological saline solution, the scaffolds were manually pressed into the bone tunnel according to the groupings mentioned.

### 4.16. Micro CT Analysis

Eight weeks after surgery and implantation of the neat PCL and PCL/5% wt. npW scaffolds into the bones, the distal femurs in the study groups were subjected to a microfocus X-ray computed tomography system (LOTUS-NDT, Behin Negareh Co., Tehran, Iran) to evaluate the ultrastructure of the implant region. The X-ray tube voltage and its current were set to 50 kV and 50 μA, and 50 kV and 60 μA. More than 1000 images of the piece were obtained and reconstructed with a nominal spatial resolution of 7 μm from the axial and 3D view. All the protocol settings processes were controlled by LOTUS NDT-ACQ software. The acquired 3D data was reconstructed using LOTUS NDT-REC by a standard Feldkamp, Davis, Kress (FDK) algorithm [74]. Moreover, LOTUS NDT-3D Image J and MATLAB software were used for rendering reconstructed images. A volume of interest (VOI) (3 mm in diameter and 10 mm in length) was co-centrically positioned over the CD site. The sample section considered contained scaffold portions and newly formed bone, as well as soft tissue portions. Therefore, thresholding was used to segment the VOI for the different portions. From this, the number of voxels relating to newly formed bone was determined, giving the BV value. The value of the bone volume fraction (BVF) in the VOI was calculated by the ratio BV/TV, where TV represents the total volume of regenerated bone, the pores enclosed in it, and the soft tissue.

### 4.17. Protein Preparation and Western Blot Analysis

Nonidet P40 (NP40) lysis buffer containing 50 mm Tris, pH 7.4, 250 mm NaCl, 5 mm EDTA, 50 mm NaF40, 1 mm Na_3_VO_4_, 1% NP40 0.02% NaN_3_ and additionally 1 mm PMSF and a specific protease inhibitor cocktail, was used to lyse the distal femur bone. The extractions were incubated on ice for 30 min, SDS-polyacrylamide gel electrophoresis (10%) was used for separation. The samples were transferred to PVDF membranes and blocked with 5% casein for 1 h. Immunoblotting was performed by incubating the membranes overnight at 4 °C with primary antibodies against Runx2, Osx, and Col1A1. Membranes were washed three times with Tris-buffered saline with Tween20 (TBST) and were incubated with horseradish peroxidase-modified secondary antibodies, and blots were then developed using ECL advanced Western blotting detection kit (Cytomatingene, Isfahan, Iran). The signals from each protein band were normalized against the Gapdh (Glyceralde-3 phosphate dehydrogenase) amount quantified by using the polyclonal anti-GAPDH antibody.

### 4.18. Statistical Analysis

Data were reported as mean ± SD, and the graphs were plotted using GrafPad Instat 3 (Microsoft Software, San Diego, CA, USA) were statistically analyzed using analysis of variances (ANOVA) followed by a post-Tukey test, and a *p*-value less than 0.05 was considered to have a significant difference.

## 5. Conclusions

Here we showed the feasibility of using the PCL/npW composite as a bone repair biomaterial. The addition of npW improved cell viability and biocompatibility due to modifying physicochemical parameters. Our results suggest that fabrication of PCL/npW composite by 3D-printing technology significantly decreased the mismatch between the scaffold and target bone at the defect site. Additionally, in vivo experiments showed that the PCL/npW composite promoted osteoblast differentiation and HA accumulation, suggesting its ability to promote new bone formation. Therefore, PCL/npW as a bone repair biomaterial would be a better scaffold with a good degradability, biocompatibility, and osteogenic differentiation capability with a high potential for applications in bone diseases and tissue regeneration. Ceramic-based composites will be a promising biomaterial for bone tissue engineering and can result in the expected 3D dimensional scaffold as a bone graft substitute with sufficient bone behavioral properties. As the accomplishment of functionality of tissue engineering techniques critically depends on the composite biomaterials, the path of formulate products have a great effect on improving the lifestyle and life expectancy of the patients. Such remarkable innovations are to be performed on the ceramic-based composite biomaterial for further biophysiochemical implications.

## Data Availability

Data and materials are available upon written request to the corresponding author.
